# Successful surgery localized to the infected lesion as diagnosed by ^18^F-fluorodeoxyglucose positron emission tomography/computed tomography for extended-aortic prosthetic graft infection

**DOI:** 10.1016/j.ijscr.2019.05.015

**Published:** 2019-05-11

**Authors:** Takasumi Goto, Kazuo Shimamura, Toru Kuratani, Keiwa Kin, Takayuki Shijo, Kenta Masada, Yoshiki Sawa

**Affiliations:** a Department of Cardiovascular Surgery, Osaka University Graduate School of Medicine, Osaka, Japan; b Department of Minimally Invasive Cardiovascular Medicine, Osaka University Graduate School of Medicine, Osaka, Japan

**Keywords:** Prosthetic graft infection, Localized surgery, ^18^F-fluorodeoxyglucose positron emission tomography/computed tomography imaging

## Abstract

**Introduction:**

We describe the successful treatment of a patient with extended-aortic prosthetic graft infection (PGI) by surgery limited to the infected lesion based on ^18^F-fluorodeoxyglucose positron emission tomography/computed tomography (^18^F-FDG-PET/CT) findings.

**Presentation of case:**

A 54-year-old man, who had undergone three graft replacements (GRs) for extended-thoracic aortic aneurysms, was diagnosed with PGI complicated by an aorto-esophageal fistula. On the basis of ^18^F-FDG PET/CT findings, we performed a redo total arch replacement, preserving the other prosthesis where abnormal FDG uptake was not detected. All the resected tissues were positive for gram positive coccus. There were no signs of infection recurrence at 2 years postoperatively.

**Discussion:**

Since activated inflammatory cells such as macrophages uptake FDG, FDG-PET/CT clarifies the localization of the infected prosthesis precisely.

**Conclusion:**

Surgery localized to the infected sites detected by FDG-PET/CT can be an effective option for PGI in cases with previous multiple GRs.

## Highlights

• Radical surgical treatment for prosthetic graft infections is still challenging.
• Especially so for patients with extended-aortic prosthetic graft infection.
• Redo total arch replacement while preserving prostheses with no abnormal FDG uptake.
• All the resected tissues were positive for methicillin-resistant *Staphylococcus epidermidis*.
• No signs of infection recurrence at 2 years postoperatively.

## 1 Introduction

Surgical treatment for prosthetic graft infections (PGI) essentially requires complete graft resections and vascular reconstructions. In the patients with extended aortic graft replacements (GRs), these radical procedures are often complicated, leading to poor postoperative prognosis [1]. In such cases, precise detection of the infected site could allow physicians to perform localized surgery on the infected sites alone.

^18^F-fluorodeoxyglucose positron emission tomography/computed tomography (^18^F-FDG-PET/CT) is useful for identifying the infected site in various infections, including PGI [2,3]. Additionally, in the patients with fever of unknown origin, FDG-PET/CT reportedly detects the infection more precisely than Ga-scintigraphy [4].

Here, we describe the successful treatment of a patient with PGI complicated by aorto-esophageal fistula (AEF) who had multiple GRs in the past for extended-TAAs by surgery limited to the infected lesion based on FDG-PET/CT findings One of the interesting points in this case is also that FDG-PET/CT could clarify localization of the infected sites more clearly than Ga scintigraphy We reported this case in line with the SCARE criteria [5].

## 2 Presentation of case

A 54-year-old man was referred to our department with fever of unknown origin. The patient had undergone three GRs for extended TAAs previously. Ten years ago, GR of the ascending aorta was performed for Stanford type-A aortic dissection. Total arch replacement (TAR) was performed for an arch aneurysm 7 years ago. Additionally, thoracic endovascular aortic repair (TEVAR) was performed for descending aortic aneurysm using two stent grafts 2 months ago.

On admission, laboratory data showed elevation of white blood cell count (WBC) and serum C-reactive protein (CRP) level (9820/μL and 4.6 mg/dL, respectively). Contrast computed tomography (CT) imaging revealed small amounts of air within the arch aneurysm. Subsequent upper gastrointestinal endoscopy demonstrated fistula formation in the mid-section of the intrathoracic esophagus. Thus, the patient was diagnosed with PGI complicated by AEF.

**Fig. 1:**
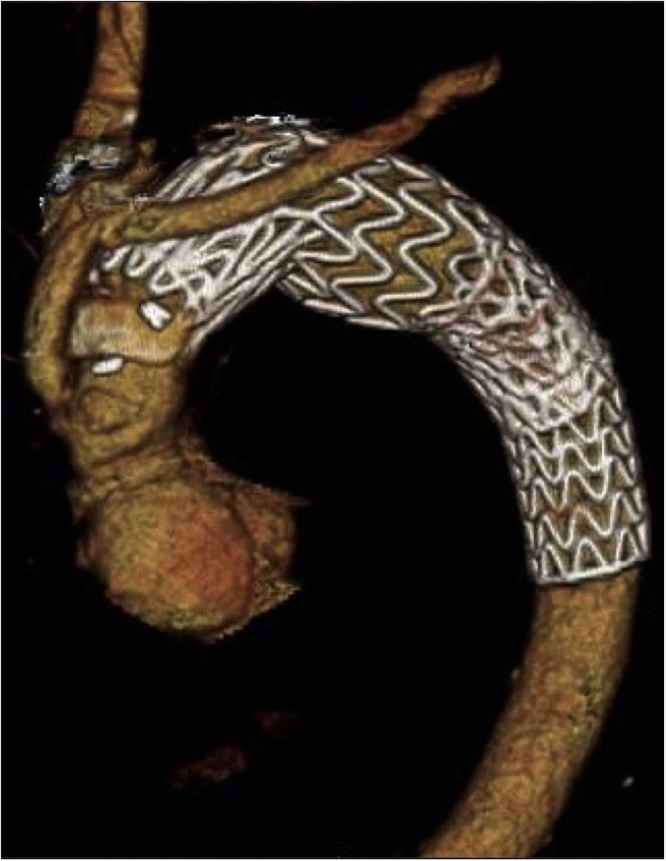
Preoperative computed tomography 3D-imaging.

Considering his relatively young age, we initially planned a radical redo-surgical treatment. To remove all prostheses, redo-GRs of the ascending aorta, aortic arch, and descending aorta with redo sternotomy and left thoracotomy were required (Fig. 1). On the basis of CT findings, a distal stent graft on the descending aorta reached at the Th 9 level. Esophageal resection and reconstruction were also required for AEF.

**Fig. 2:**
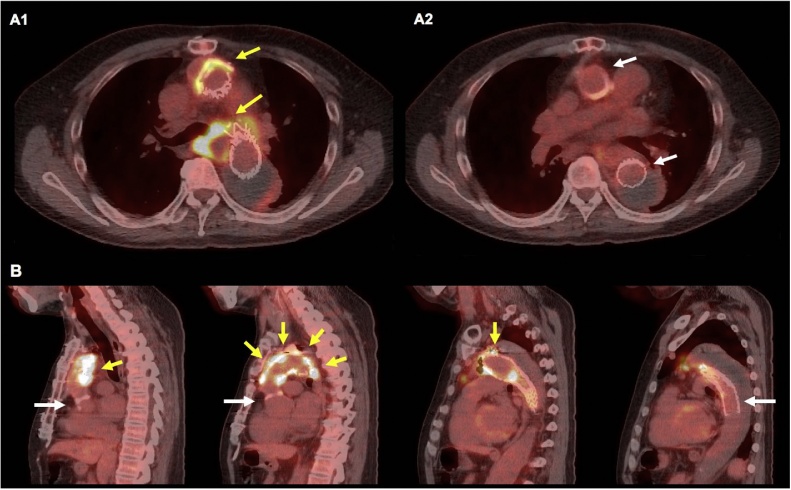
Preoperative ^18^F-fluorodeoxyglucose positron emission tomography/computed tomography (^18^F-FDG-PET/CT) findings (A; axial, B; sagittal view). Significant FDG uptake at the lesions with yellow arrows, while abnormal uptake was not observed at the sites with white arrows.

**Fig. 3:**
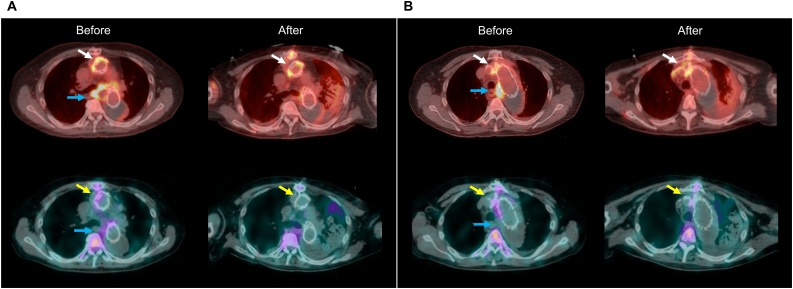
Comparison between FDG-PET/CT and Ga scintigraphy after esophagectomy. Blue arrows show abnormal uptake of each tracer around aorto-esophageal fistula. White arrows show abnormal uptake of FDG in the infected lesions whose cultures were positive for methicillin-resistant Staphylococcus epidermidis, and yellow arrows abnormal uptake of Ga in these same lesions (A; distal anastomosis of the ascending graft, B; aortic arch). After esophagectomy, there were no significant Ga uptake at those infected lesions, while high FDG uptake remained at those infected lesions (white and yellow arrows).

As an alternative, we evaluated whether localized surgical treatment of the infected lesions is feasible. To evaluate the infected sites, FDG-PET/CT and Ga scintigraphy were performed. On FDG-PET/CT, abnormal FDG uptakes were found at the following three sites: the distal anastomosis of the ascending graft, the aortic arch aneurysm, and the AEF (Fig. 2). FDG uptake was absent at the proximal parts of the ascending graft and the descending endo-graft. Whereas, on Ga scintigraphy, abnormal Ga uptakes were observed at the same three sites, but with the uptake in each of these lesions being clearer on FDG-PET/CT than on Ga scintigraphy (Fig. 3).

Prior to redo-GRs, we performed the transthoracic esophagectomy using the right video-assisted thoracoscopic surgery approach for AEF. FDG-PET/CT and Ga scintigraphy were performed again after esophagectomy. Although abnormal FDG uptake remained at the distal anastomosis of the ascending graft, in contrast, there were no significant Ga uptakes (Fig. 3A). Based on FDG-PET/CT findings, we planned a redo-TAR, preserving the previous grafts with no significant FDG uptakes.

**Fig. 4:**
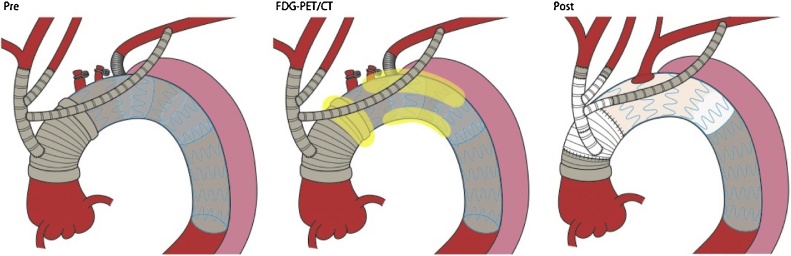
Details of the redo-total arch replacement (TAR) with omental flap procedure. The lesions indicated in yellow show abnormal FDG uptake. The parts indicated in white show the reconstructed grafts, while the parts in grey show the preserved prosthesis.

Three weeks after esophagectomy, redo-TAR was performed. After general anesthesia, redo sternotomy was performed. The infected site at the distal part of the ascending graft was confirmed visually. After cardiopulmonary bypass (CPB) establishment with systemic heparinization, systemic cooling was performed until the deepest rectal temperature was <28 ℃. Maintaining cerebral perfusion by CPB through the right axillary artery, the bypass grafts of brachiocephalic artery and left common carotid artery were resected and reconstructed (Fig. 4). Cardiac arrest was achieved by occluding the aorta using an occlusion balloon through the femoral artery. Based on FDG-PET/CT findings, the distal part of the ascending graft and 2 endografts at the aortic arch were removed, leaving the proximal part of the ascending graft and the descending endograft. Subsequently, redo-TAR was performed using the frozen elephant trunk technique with CTAG (GORE, 31-31-15). The fully-reconstructed graft was covered with an omental flap. The durations of circulatory arrest, aortic cross-clamp, and CPB were 4, 186, and 305 min, respectively.

Six weeks after the redo-TAR, the reconstruction of the esophagus was performed using an ileocolic conduit through the ante-thoracic route. All cultures of the removed prosthesis were positive for methicillin-resistant *Staphylococcus epidermidis* (MRSE). Appropriate anti-biotic therapy using vancomycin was continued pre- and post-operatively. The postoperative course was uneventful. The patient underwent CT and laboratory tests regularly, but there were no signs of recurrent infection or re-increase of inflammatory markers in the 2 years following the surgery.

## 3 Discussion

Complete resection of all the prostheses is often performed for PGI. However, some patients with PGI are reportedly successfully treated by only drainage of the infected sites [6]. These findings suggest that, to control the inflammation, it is crucial to reduce the bacterial load to a level that can be controlled by the host immune system. Particularly for high-risk patients with extended-aortic graft replacement, a limited surgery focused on the infected sites could help reduce the surgical risk, leading to improvement of postoperative prognosis.

Precise detection of the infected sites may offer physicians the option to perform localized surgery on the infected site alone. Tokuda et al. reported two cases of PGI diagnosed by FDG-PET/CT, who had previous GRs for TAA and abdominal aortic aneurysms [3]; since significant FDG uptake was not observed in the abdominal graft, redo-GR was performed only for the thoracic graft. No recurrences were observed within 1 year postoperatively. However, our case had grafts consistently from the ascending aorta to the distal descending aorta, and we resected only a part of the prosthesis, which has not been reported before.

Our case also suggests that, in PGI, FDG-PET/CT could clarify the localization of the infected sites more clearly than Ga scintigraphy. During the 2^nd^ FDG-PET/CT after esophagectomy, abnormal FDG uptakes were detected in the distal anastomosis of the ascending graft, whose cultures were positive for MRSE. The same sites showed no Ga uptake (Fig. 3). Generally, the sensitivity of FDG-PET/CT is superior to that of Ga scintigraphy [4]. Given that all infected prostheses must be resected during localized surgery for PGI, FDG-PET/CT is more useful than Ga scintigraphy in such cases.

## 4 Conclusion

Surgery localized to the infected sites detected by FDG-PET/CT can be an effective option for patients who have undergone several previous GRs for extended-TAAs.

## Conflicts of interest

None.

## Sources of funding

None.

## Ethical approval

This presentation is approved by ethical committee of our hospital.

## Consent

Informed consent was obtained from the patient in written and verbal form.

## Author contribution

Takasumi Goto wrote this manuscript, collecting all clinical data. Kazuo Shimamura proofread this case report. Toru Kuratani supervised this study. Keiwa Kin, Takayuki Shijo and Kenta Masada performed or assisted all the procedures. Yoshiki Sawa also supervised this report.

## Guarantor

Yoshiki Sawa has full responsibility of this case.

## Provenance and peer review

Not commissioned, externally peer-reviewed.
